# Metabolic alterations of the immune system in the pathogenesis of autoimmune diseases

**DOI:** 10.1371/journal.pbio.3002084

**Published:** 2023-04-25

**Authors:** Luz P. Blanco, Mariana J. Kaplan

**Affiliations:** Systemic Autoimmunity Branch, National Institute of Arthritis and Musculoskeletal and Skin Diseases, National Institutes of Health, Bethesda, Maryland, United States of America

## Abstract

Systemic autoimmune diseases are characteristically associated with aberrant autoreactive innate and adaptive immune responses that lead to tissue damage and increased morbidity and mortality. Autoimmunity has been linked to alterations in the metabolic functions of immune cells (immunometabolism) and, more specifically, to mitochondrial dysfunction. Much has been written about immunometabolism in autoimmunity in general, so this Essay focuses on recent research into the role of mitochondrial dysfunction in the dysregulation of innate and adaptive immunity that is characteristic of systemic autoimmune diseases such as systemic lupus erythematosus (SLE) and rheumatoid arthritis (RA). Enhancing the understanding of mitochondrial dysregulation in autoimmunity will hopefully contribute to accelerating the development of immunomodulatory treatments for these challenging diseases.

## Abstract 2

Systemic autoimmune diseases are linked to alterations in the metabolic functions of immune cells. This Essay addresses key aspects of the current knowledge on how mitochondrial dysfunction in particular affects human autoimmune diseases.

## Introduction

The main function of the human immune system is to protect us against microbes, cancer cells, and other threats through its 2 arms: the innate and adaptive immune systems (Box 1). The immune system requires a delicate and active process that balances antimicrobial effector functions with adequate mechanisms to resolve inflammation and prevent self-damage. Indeed, dysregulated immune responses and an imbalance in these mechanisms can lead to tissue damage, inflammation, and autoimmunity. The term “autoimmunity” covers a myriad of conditions in which the immune system synthesizes or fails to clear antibodies and immune cells that are reactive against self-antigens (also known as autoantigens), triggering damage of the targeted tissues and specific diseases. The road to autoimmunity is complex, multifactorial, and comprises many putative steps involving complex crosstalk between various immune and stromal cells; it is influenced by environmental exposures, genetic and epigenetic factors, and poorly understood stochastic events [1,2]. The metabolism of immune cells is crucial for the regulation of immune activation in homeostasis and disease states. Indeed, metabolism regulates cellular aspects such as proliferation, differentiation, cell fate, and effector functions of immune cells because all these processes depend on proper energy supplies. In addition, cellular metabolism not only regulates energy fluxes but also can affect gene expression by epigenetic modifications (Box 1), adding another layer of complexity to the immunopathogenesis and possible therapeutic targets in autoimmune diseases [3].

In this Essay, we discuss recent findings related to changes in the metabolic functions of immune cells (immunometabolism), particularly mitochondrial dysfunction, in innate and adaptive immune cells and their contribution to the generation of autoimmunity, with emphasis on systemic lupus erythematosus (SLE) and rheumatoid arthritis (RA) as examples of systemic autoimmune conditions (Box 1).

**Box 1. Glossary**

**Innate immune system**

The body’s first line of defense, quick in its responses and generally nonspecific. The main cellular components are myeloid cells, including granulocytes (neutrophils, eosinophils, and basophils), monocytes, macrophages, and dendritic cells (DCs), but other cells such as gamma delta T cells, innate lymphoid cells (ILCs), and mast cells can be considered part of the innate immune system. Innate immune cells are the first in line to directly detect and effectively destroy threats: endowed with a powerful broad range of antimicrobial molecules and functions, including phagocytic capacity, antigen presentation, synthesis of enzymes, vasoactive factors, pore-forming proteins, chemokines, and cytokines.

**Adaptive immune system**

Mostly consists of B cells and T cells, which have powerful and specific mechanisms to destroy and remember pathogens for future encounters, making them essential for vaccine effectivity and immunity against pathogens.

**Epigenetic modifications**

A series of chemical modifications of nucleic acids and/or histones to modulate gene expression without changing the genetic code. Some of the modifications include methylation, acetylation, and the recently described lactylation.

**Immunologic tolerance**

An active process of the immune system that deals with preventing or reverting antigen-specific autoreactive responses.

**Systemic lupus erythematosus**

An autoimmune syndrome characterized by multi-organ damage due to inflammation. Systemic lupus erythematosus (SLE) is characterized by the presence of circulating autoantibodies that recognize double-stranded DNA, histones, and a variety of RNA–protein complexes, among other autoantigens. The formation of immune complexes leads to proinflammatory and pro-oxidative environments and tissue damage.

**Rheumatoid arthritis**

Rheumatoid arthritis (RA) is a chronic inflammatory disease in which the synovial joints are targeted by autoantibodies and autoreactive immune cells. In severe cases, the inflammation can also affect other tissues including the heart, lungs, eyes, nerves, and skin.

**Inflammasome**

A complex intracellular structure formed by diverse components that assemble together in response to inflammation, oxidative stress, and infectious triggers.

**Antigen presentation**

Required to avoid self-recognition and the development of autoreactive immune responses, and to generate enduring protective immunological memory. Antigen-presenting cells (APCs) internalize and process microbes and antigens. Upon processing, the ingested antigens are transformed into peptides. Peptides are presented at the APCs’ membrane by major histocompatibility complex (MHC) class II molecules. Specific T cells recognize the presented MHC–peptide complex, becoming activated. Activated T cells proliferate and engage through their T-cell receptor with the respective antigen-specific B cell (which present the correct membrane-associated MHC–peptide complex) promoting their transition into plasma cells, which synthetize specific antibodies.

## Autoimmunity and metabolism

The mechanisms by which innate immune cells promote autoimmune responses have become significantly clearer over the past few decades. Overactive innate immune cells responding to microbial triggers or other danger signals might cause tissue damage and undergo or promote inflammatory cell death, generating and modifying endogenous danger-associated molecular patterns (DAMPs), autoantigens or neoantigens [4]. This autoantigen modification may be triggered by a variety of mechanisms, including enhanced inflammatory oxidative stress involving the synthesis of reactive oxygen species (ROS) that can modify nucleic acids, lipids, carbohydrates, and proteins. In predisposed hosts, these neoantigens can contribute to fueling the fire and promote downstream inflammatory responses. Dead cells and cellular debris that are not cleared promptly by innate immune cells can expose modified nuclear and mitochondrial-derived DNA, as well as RNA, that can have potent proinflammatory effects on target cells and activate toll-like receptors (TLRs) and other membrane-bound and cytoplasmic sensors of nucleic acids and associated proteins [4] (Box 2).

**Box 2. Key points in the generation of autoimmunity and mitochondrial dysfunction**

➢ Genetic propensity, environmental stimuli, and stochastic events trigger the generation of proinflammatory and pro-oxidative pathways.
➢ In this milieu, inflammatory cell death and improper clearance of dead cells can trigger and perpetuate neoantigen generation and inflammation.
➢ Dysfunctional mitochondrial function is prevalent and perpetuates in inflammatory conditions and under hypoxia.
➢ Mitochondrial dysfunction affects cells’ metabolism, perturbing regulatory networks, fueling proinflammatory responses, and interfering with immune system homeostasis.

Regarding the adaptive immune system, autoreactive T cells can escape the process of thymus education in which they are eliminated before accessing the periphery (areas outside the primary lymphoid organs) [5], where they might cross-recognize microbial products or other danger signals. These products can mimic autoantigens, acting as neoantigens that can lead the adaptive immune cells to trigger unintended autoreactive responses [6]. Additionally, failure in the generation and/or function of regulatory T (T_reg_) cells and regulatory B (B_reg_) cells, key in the development of immunologic tolerance (Box 1), may also be involved in the induction of autoimmune responses [7]. Immune tolerance responses encompass the development and engagement of specific tolerogenic cellular and humoral responses to retain and also expand mechanisms of immune regulation [8]. Akin to inducing an antigen-specific protective immune response, many steps can go awry and disturb the development of tolerance, promoting autoreactive and autoimmune responses. Indeed, several approaches to promote and restore tolerance have been investigated, including desensitization strategies, oral immunotherapy, HLA desensitization, immunoablation followed by hematopoietic stem cell transplant, as well as vaccines designed to induce tolerogenic responses [9–11].

The development of these opposing (inflammatory versus tolerogenic) immune responses and cellular networks requires specific metabolic responses. Effective immune responses entail heightened cellular metabolism, biosynthetic potential, and cellular division. A shared feature of both inflammatory and cancer cells is a metabolic reprograming activity required for actively dividing cells. This metabolic reprograming in cancer cells, known as the “Warburg effect,” in which cells increase the rate of glucose uptake and preferential production of lactate, takes place in cells that are actively dividing and that have increased demand for biosynthetic molecular precursors and enzyme cofactors [12].

Another shared feature between inflammatory and cancer cells is that they function in hypoxic microenvironments in the tissues they infiltrate. Hypoxia development is frequently observed in inflamed tissues in autoimmune diseases [13] and is a stressor for mitochondria that triggers the electron transport chain to work in reverse flow. As such, instead of producing adenosine triphosphate (ATP), it generates mitochondrial ROS (mROS) that can further contribute to oxygen consumption. Furthermore, hypoxia promotes the use of fumarate instead of oxygen as the terminal electron acceptor [14,15]. Under hypoxic and/or inflammatory conditions, mitochondria are not fully functional and display impaired ability to provide nicotinamide adenine dinucleotide (NAD^+^) coenzyme, leading to activation of alternative cellular metabolic cycles such as glycolysis to provide both ATP and NAD^+^ [16]. Due to increased glycolytic activity, acidification may ensue through enhanced lactic acid generation. Cellular damage can then be quickly amplified due to further promote mitochondrial dysfunction.

It is important to consider that these metabolic pathway adjustments are essential for the survival of cells and to effectively fight infections; therefore, they cannot be completely blunted. As such, it is imperative to learn in more detail how to harmonize the balance among these pathways to avoid aberrant inflammatory responses while mounting an adequate antimicrobial response (Box 2).

## Mitochondrial dysfunction and autoimmunity

### Genetic associations

The genetic factors involved in the common susceptibility to both autoimmune diseases and mitochondrial dysfunction remain incompletely characterized. Potentially highlighting a critical role for enhanced mROS, and common to both RA and SLE, are genetic variants in major histocompatibility complex (MHC) class II molecules that can lead to enhanced mROS production in antigen-presenting cells (APCs). Through a noncanonical mechanism that seems to be independent on specific antigen–T cell interactions, but which relies on the recognition of certain inflammation-derived DAMPs by specific sequences (epitopes) on MHC class II molecules, a signaling pathway is activated that promotes enhanced mROS activity and mitochondrial dysfunction in the APCs [17]. Additionally, genetic alterations in reduced nicotinamide adenine dinucleotide phosphate (NADPH) oxidase enzymatic complex components may lead to enhanced mROS production in low-density granulocytes (LDGs; a type of proinflammatory neutrophil) from patients with SLE and in chronic granulomatous disease [18]. This hyperproduction of mROS may be a compensatory mechanism to overcome deficient function of the NADPH machinery in these cells.

### Innate autoimmune mechanisms and mitochondrial dysfunction

#### Neutrophil-associated mechanisms

**Fig 1:**
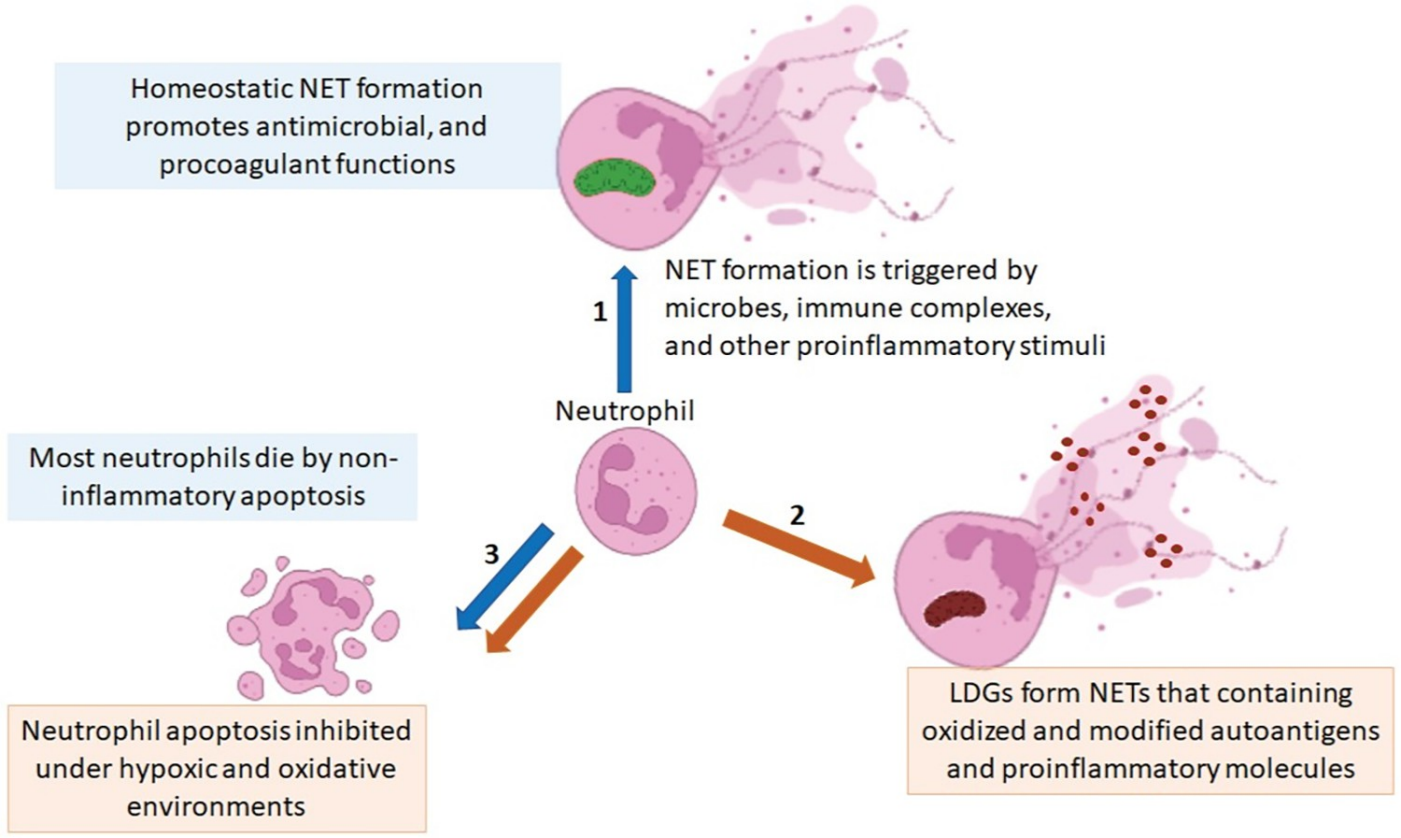
Neutrophils and NET formation in autoimmunity. Certain proinflammatory signals or microbes induce neutrophils to form NETs (blue arrow #1). Chronic proinflammatory or other unknown stimuli generate LDGs with enhanced ability to synthesize mROS and form NETs (brown arrow #2). LDG-derived NETs are enriched in proinflammatory and interferogenic molecules. Neutrophils are actively recycled by apoptosis, so proinflammatory molecules and neoantigens need to be promptly cleared (brown arrow #3). As such, apoptosis is an important mechanism to avoid activated proinflammatory neutrophil persistence (blue arrow #3). Figure prepared using Biorender’s templates. LDG, low-density granulocytes; mROS, mitochondrial reactive oxygen species; NET, neutrophil extracellular trap.

Neutrophils are the most abundant cells in human blood and can act as important sources of extracellular mitochondrial and nuclear-derived proinflammatory nucleic acids/autoantigens. This externalization is driven in part by the ability of these cells to form neutrophil extracellular traps (NETs). NET formation is triggered by a variety of infectious and sterile proinflammatory stimuli and leads to the externalization of nuclear and mitochondrial DNA (mDNA) and various RNAs bound to histones and other proteins [18–20]. While NET formation appears to have important homeostatic, antimicrobial, and procoagulant functions (Fig 1) [21], the NET formation process can go awry in SLE and other autoimmune diseases. Certain autoantigenic immune complexes can drive neutrophils to synthesize enhanced levels of mROS, leading to increased oxidation of the mDNA that gets extruded in the NETs and induces proinflammatory responses on target cells [18]. In addition, in SLE, there is a subset of neutrophils termed LDGs that can spontaneously form NETs and have increased mROS synthesis (Fig 1) [18,22,23]. These neutrophils extrude oxidized nucleic acids that promote induction of type I interferons (IFNs; cytokines crucial in SLE pathogenesis) in target cells, primarily through the cyclic GMP-AMP synthase (*cGAS*)–stimulator of interferon genes (*STING*) pathway and/or through endosomal TLRs [18,19,24]. Other mitochondria-derived components (such as N-formyl peptides) may also contribute to neutrophil activation and perpetuate chronic inflammatory responses in RA and other conditions [25].

Importantly, other cell death mechanisms besides NET formation have recently been identified as putative contributors to SLE pathogenesis [26]. Ferroptosis, in which lipid peroxidation occurs via iron-dependent oxidative mechanisms, affects mitochondria [27], inducing mROS synthesis. The mROS produced may trigger pore formation and depolarization in the mitochondrial membrane via oligomerization of mitochondrial voltage dependent anion channel-1 (VDAC-1) [28]. Increased mROS synthesis can extend the half-life of neutrophils by inhibiting apoptosis through stabilization of the hypoxia master transcriptional regulator HIF-1α (Fig 1) [29]. Through mROS-induced VDAC pores, fragments of mDNA can reach the cytoplasm and further stimulate type I IFN production and proinflammatory pathways [28].

#### Myeloid antigen presenting cell-associated mechanisms

**Fig 2:**
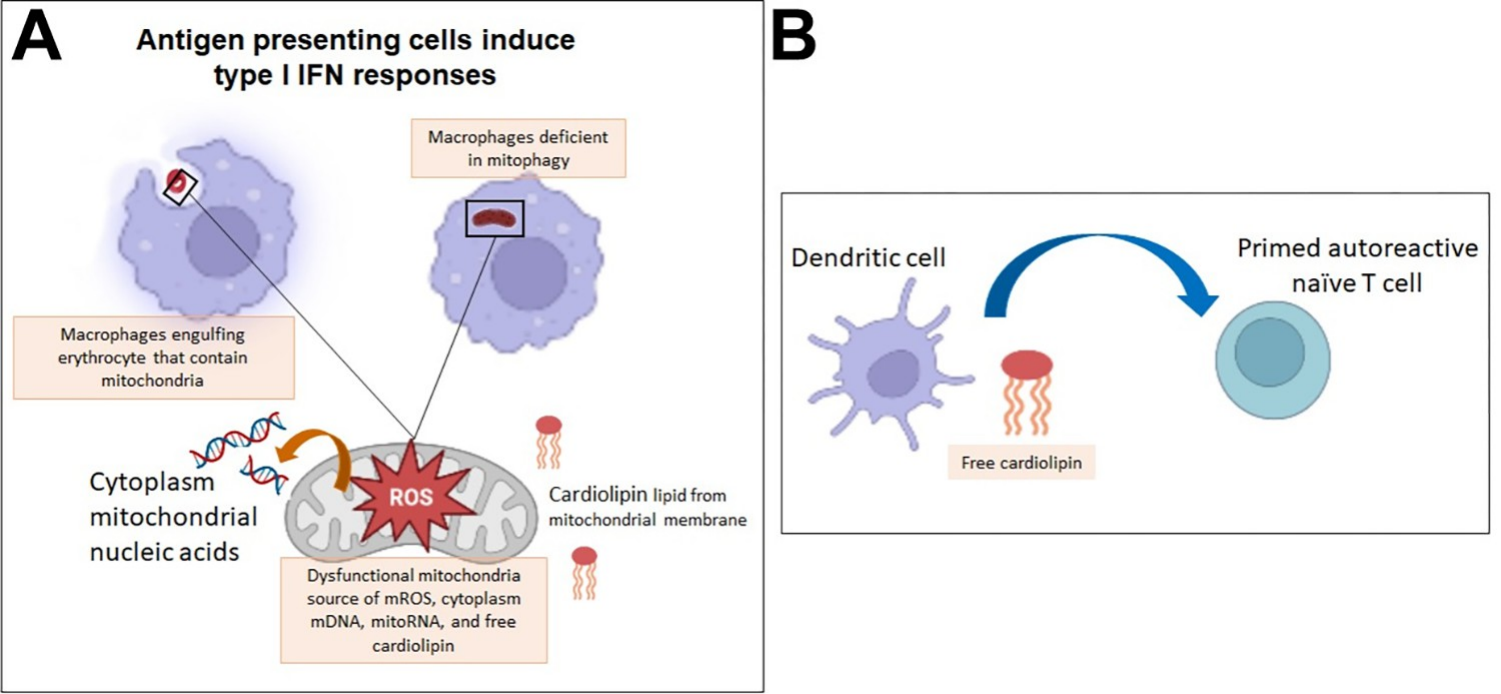
How myeloid cells with dysfunctional mitochondria contribute to autoimmunity. (A) Type I interferon-generating events in myeloid APCs are the internalization of erythrocytes that contain mitochondria and mitophagy deficiency in APCs. Both events allow mDNA and mRNA into the cytoplasm, triggering cGAS–STING and TLR-sensing systems and activation of the type I IFN pathway. (B) Dying myeloid APCs release cardiolipin, a lipid component of the mitochondrial membrane, in addition to mitochondrial nucleic acids. Free cardiolipin is a potent adjuvant that might allow DCs to prime autoreactive naïve T cells. Figure prepared using Biorender’s templates. APC, antigen-presenting cell; DC, dendritic cell; IFN, interferon; mDNA, mitochondrial DNA; TLR, toll-like receptor.

Myeloid APCs, including macrophages, monocytes, and dendritic cells (DCs), are considered important contributors to autoimmunity, and their metabolism is also reprogramed in inflammatory conditions. Indeed, mitochondrial quality control in myeloid cells is a key anti-inflammatory mechanism [30]. Mitophagy is the process by which old/dysfunctional mitochondria are discarded from the cell and is central to mitochondrial quality control. When mitophagy is disrupted in myeloid cells, mDNA can reach the cytoplasm and activate nuclear sensing pathways including cGAS–STING, leading to increased type I IFN synthesis [31] (Fig 2A). Another way that these pathways can be triggered in myeloid cells is through the internalization of aberrant externalized mitochondria and mDNA (Fig 2A). In SLE, a subset of patients show evidence that their erythrocytes do not fully differentiate and therefore retain their mitochondria and mDNA [32], which can be internalized by macrophages (or other target cells) during homeostatic clearance of the erythrocytes. Furthermore, cardiolipin (a mitochondrial membrane phospholipid) is a potent adjuvant for myeloid APCs that can potentially prime autoreactive naïve T cells and thereby contribute to the development of aberrant adaptive autoimmune responses [33] (Fig 2B).

Another mechanism of mROS-triggered myeloid cell death during infection and inflammation is pyroptosis, which is a lytic process induced in inflammasome-activated cells to counteract inflammatory processes. Once activated, the inflammasome (Box 1) leads the cells to acquire pores in the cell membrane that allow the secretion of synthesized proinflammatory cytokines such as active IL-1β and IL-18, perpetuating the inflammatory process [34]. In SLE, inflammasome activity is perturbed and, together with increased mROS production, might contribute to the pathogenesis of the disease through increased generation of autoantigens by pyroptosis and enhanced synthesis of proinflammatory cytokines [35].

Overall, disruptions in mitochondrial function and immunometabolism seem to be a driving factor that affects the function of myeloid cells, promoting proinflammatory and autoimmune responses in SLE, RA, and other conditions. Promising preclinical evidence (see section on Modulation of mitochondrial dysfunction) indicates that targeting dysfunctional immunometabolism may have therapeutic benefits in these diseases.

#### Autophagy in innate immune cells

**Fig 3:**
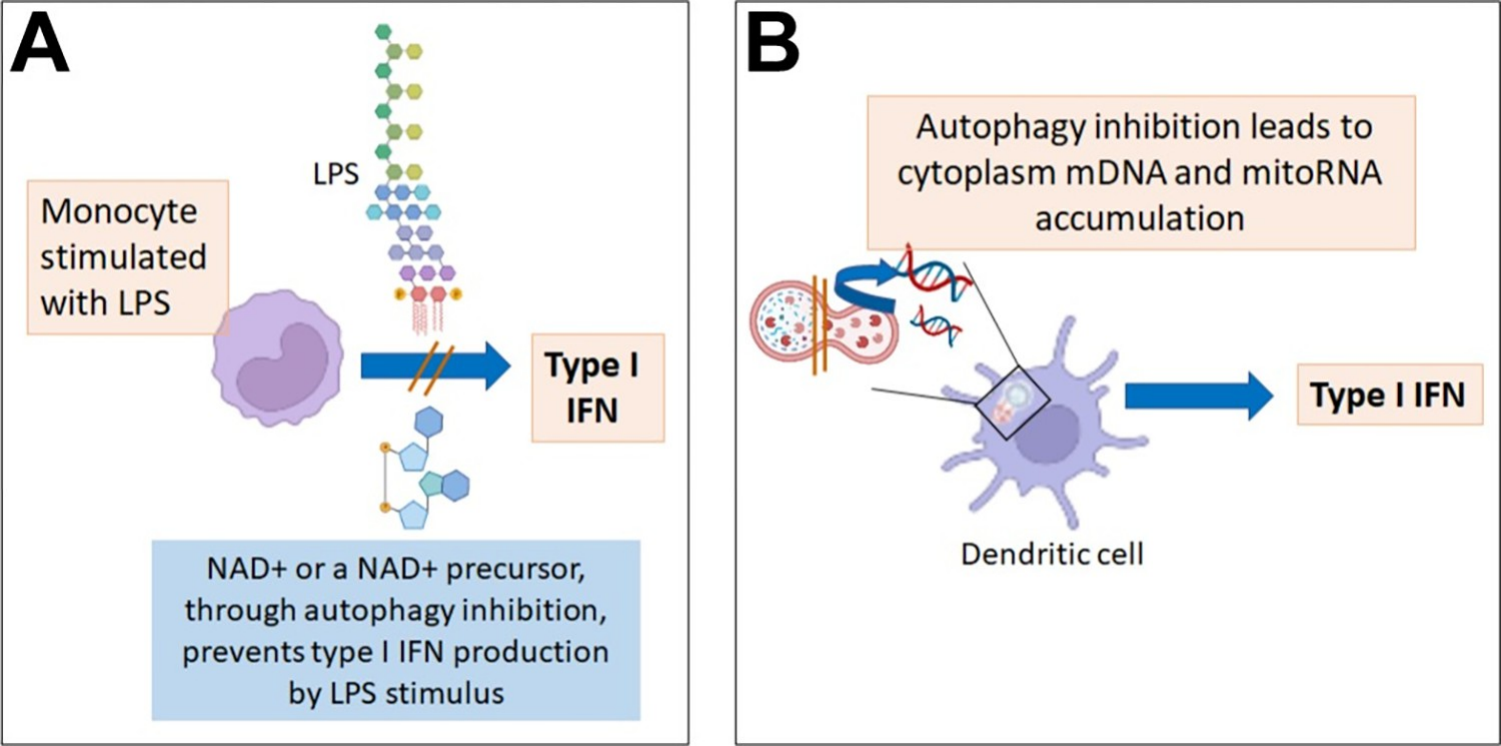
Autophagy modulating the interferon pathway. (**A**) The induction of type I IFN responses by LPS in monocytes is blunted by NAD^+^ precursor autophagy inhibition. (**B**) By contrast, autophagy inhibition in DCs blunts mDNA and mRNA removal in the cytoplasm, leading to type I IFN pathway activation and priming of autoreactive T cells. Figure prepared using Biorender’s templates. DC, dendritic cell; IFN, interferon; LPS, lipopolysaccharide; mDNA, mitochondrial DNA; NAD, nicotinamide adenine dinucleotide.

Autophagy is a self-degradative process that allows recycling of damaged/old organelles. It is critical for the proper balance of energy sources during conditions of starvation and nutrient stress. While considered a survival mechanism, it has also been linked to non-apoptotic cell death. Autophagy can also promote antigen presentation (Box 1) and the synthesis of proinflammatory cytokines. Dysregulation in autophagy has been linked to autoimmunity. Associated to immunometabolism, this pathway can be regulated by the anti-inflammatory molecule nicotinamide riboside, a precursor of NAD^+^ essential coenzyme, which blunts the secretion of lipopolysaccharide-induced type I IFN in activated healthy control or SLE monocytes [36] (Fig 3A).

The role of disruptions of autophagy in autoimmunity is complex, cell-dependent, incompletely characterized, and has potentially important implications if systemic modulation is considered. For example, contrary to the nicotinamide riboside beneficial effect in SLE monocytes, inhibition of autophagy can promote the generation of inflammatory DCs that are able to prime the autoreactive T cells that perpetuate autoimmunity. Inhibition of autophagy in this case allows mDNA to reach and remain in the DC cytoplasm, thereby promoting type I IFN responses [37] (Fig 3B). Moreover, in endothelial cells, the inhibition of autophagy enables the trans-endothelial migration of leukocytes from vasculature into tissues, which may further fuel inflammation by allowing more leukocytes to be recruited into inflamed tissues [38]. Accordingly, therapies to control these processes should consider the tissue microenvironments and the specific cellular players that need to be targeted.

### Adaptive autoimmune mechanisms and mitochondrial dysfunction

Adaptive immune cells are essential for producing strong, specific, and long-lasting immune responses. These cells and the defensive responses triggered are powerful and, as such, there are specific regulatory mechanisms both to induce their activity and to mitigate it. The relevant role of mitochondria functionality both in the normal and pathological physiology of B cells and T cells has been recently reported for a variety of diseases including SLE and RA.

#### B cell-associated mechanisms

**Fig 4:**
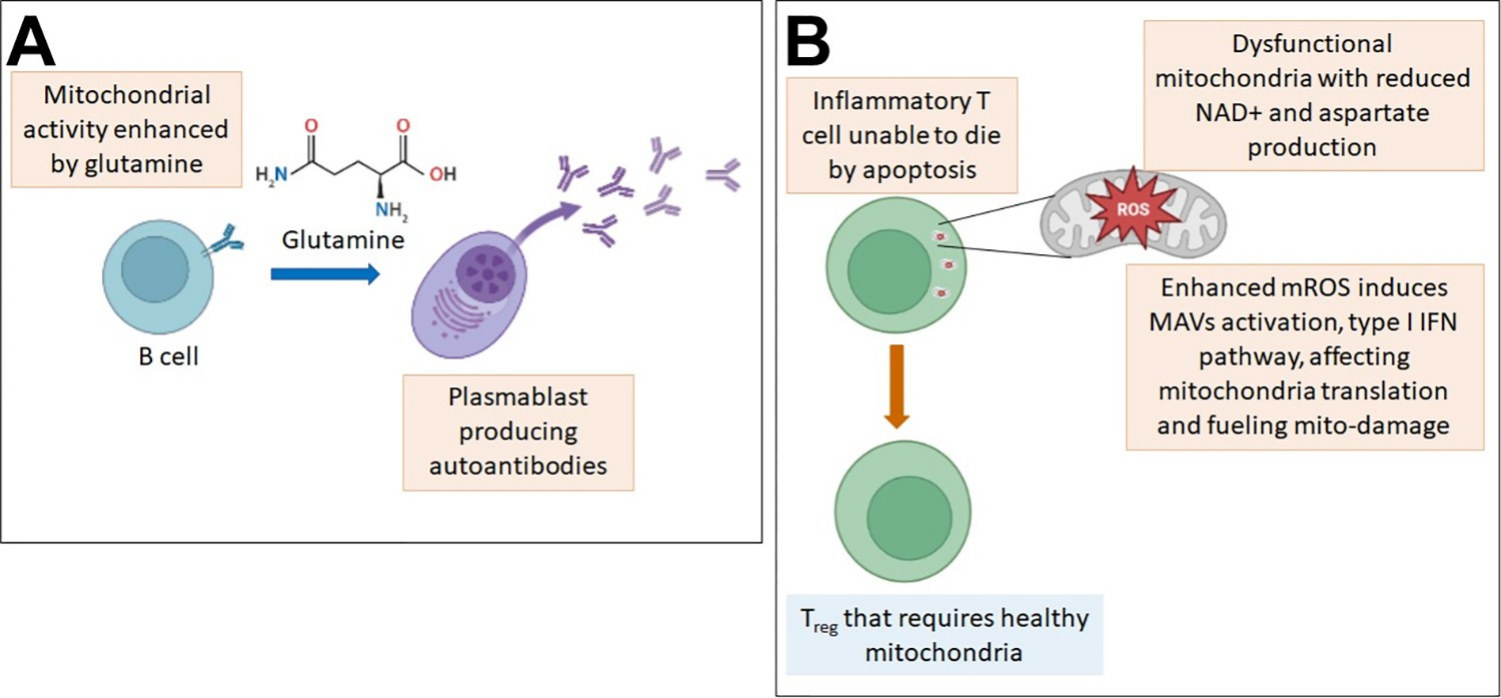
Mitochondrial dysfunction in autoimmune adaptive immune cells. (**A**) Glutamine metabolism enhances mitochondrial function in B cells fueling autoreactive plasmablast generation. (**B**) Enhanced mROS promote mitochondrial antiviral signaling protein (MAVS) activation and induction of type I IFN pathway; it also reduces T cell death, fueling inflammation. Dysfunctional mitochondria due to deficient NAD^+^ and aspartate production by mitochondria have been reported in inflammatory T cells infiltrating joints in patients with RA. The excessive mROS affects mito-translation, contributing to the pathologic cycle of mitochondrial dysfunction. In this environment, regulatory T-cell generation is impaired. Figure prepared using Biorender’s templates. IFN, interferon; mROS, mitochondrial reactive oxygen species; NAD, nicotinamide adenine dinucleotide; RA, rheumatoid arthritis.

B cells have crucial roles in the pathogenesis of systemic autoimmune diseases. Increased mitochondrial activity in SLE has been linked to enhanced differentiation of B cells into plasmablasts, with putative undesired effects linked to autoantibody production [39]. For the pathologically enhanced mitochondrial activity in B cells, glutamine metabolism is required [39] (Fig 4A). Additionally, enhanced mitochondrial activity and autoantibody production by long-lived memory B cells in SLE may be facilitated by deficient peroxiredoxin 6 (PRDX6) antioxidant activity [40] and by an intact mitochondrial cardiolipin remodeling enzyme activity provided by tafazzin [41]. In SLE, mitochondrial dysfunction might also promote the formation and externalization of mitochondria-derived autoantigens recognized by B cells, including mitofusin 1 and complement component 1q subcomponent-binding proteins [42]. Dysfunctional mitochondria that acquire somatic mDNA mutations via cumulative mROS damage may promote subsequent generation of modified self-peptides, breaching tolerance [43]. The feasibility of this mechanism is supported by the observation that, in the mitochondria, reactive mROS are synthesized near mDNA, making it an easy target for oxidation and prone to acquiring mutations.

#### T cell-associated mechanisms

T cells have a primordial role in the development of autoimmunity. T-cell activation is triggered following the recognition of antigen presented on MHC class II molecules in the membrane of APCs. T-cell activation promotes differentiation of various subclasses of T cells depending on the secondary signals received from the APCs and other innate immune cells. Once activated, T cells secrete various proinflammatory cytokines, proliferate, assist the transition of specific B cells into plasma cells and, for certain T-cell subsets, deploy cytotoxic effects onto specific cellular targets. All these activities heavily rely on their metabolic and mitochondrial activity, and various perturbations of these processes have been recently described for autoimmune conditions. For example, oligomerization of mitochondrial antiviral signaling protein (MAVS) occurs in response to viral infections and leads to type I IFN-driven antiviral responses; however, this process is perturbed in patients with SLE as a consequence of mitochondrial dysfunction, whereby enhanced mROS synthesis can directly trigger spontaneous MAVS oligomerization in lymphocytes, leading to downstream induction of the type I IFN pathway [44] (Fig 4B). Indeed, chronically activated SLE T cells with hyperpolarized mitochondria have enhanced mROS synthesis [45] and can coexist in hypoxic conditions in SLE-related glomerulonephritis and in animal models of lupus, promoting additional aberrant immune responses [46]. T cells in these tissues may display increased HIF-1α expression that interferes with their ability to die and promotes their infiltration into kidney tissue, fueling further damage [47].

In RA, proinflammatory T cells are generated in part due to an inability to renew the pool of NAD^+^ coenzyme. RA inflammatory T cells have deficient tricarboxylic acid (TCA) cycle aspartate production by dysfunctional mitochondria. Proinflammatory T cells populate the RA synovium and promote tissue damage [48] (Fig 4B). Strikingly, transferring healthy mitochondria into pathogenic RA-patient-derived T cells attenuates inflammation and reduces the production of the proinflammatory cytokine TNF [48].

The mitochondrial translation process is also important for T-cell function. When mitochondrial translation is deficient, the mitochondrial respiration machinery lacks essential proteins required for proper functioning of the electron transport chain, eventually leading to general dysfunction and enhanced mROS synthesis (Fig 4B). Of note, antibiotics that target prokaryote ribosomes may affect T-cell mitochondrial translational machinery, reducing the deleterious exacerbated mitochondrial respiration and ameliorating autoimmunity features [49].

#### Regulatory lymphocyte-associated mechanisms

Global inhibition of mitochondrial physiology is not always helpful in autoimmune diseases and can be deleterious for the function and maintenance of T_reg_ and B_reg_ cells, which are crucial for the tolerance process and long-term control of autoimmunity [50,51]. Mice deficient in estrogen-related receptor gamma gene (*Esrrg*) spontaneously develop autoimmune diseases in association with dysfunctional T_reg_ cells and mitochondrial defects [52]. Furthermore, suppressing the GDP-forming β-subunit of succinate-CoA ligase (SUCLG2), which leads to reduced mitochondrial activity, increases proinflammatory T cells in the RA synovium [53]. Autophagy and the activity of transcription factor TFEB are induced in T cells that transform into T_reg_ cells. TFEB has a critical role in this T_reg_ cell differentiation and function by improving mitochondrial fitness [51] (Fig 4B).

## Modulation of mitochondrial dysfunction

Given the link between mitochondrial dysfunction and type I IFN synthesis, it has been hypothesized that modulating or improving aberrant neutrophil mitochondrial dysfunction and myeloid bioenergetics could have beneficial effects in SLE. Indeed, mROS scavengers and inhibitors of VDAC oligomerization can attenuate lupus features in mice and normalize dysregulated neutrophil responses [18,28]. Administration of analogs of the essential electron carrier coenzyme Q, such as idebenone or MitoQ, to improve mitochondrial function also had beneficial effects on murine lupus models [54,55]. Itaconate, a TCA cycle mitochondrial-derived metabolite produced in response to inflammation, blunts oxidative stress, proinflammatory, and type I IFN-generating responses [56]. Lupus mice treated with an itaconate derivative showed significant attenuation of disease progression [57]. Furthermore, repairing the activity of oxyguanine glycosylase 1 (OGG1), an enzyme involved in excising damaged oxidized nucleic acids, attenuated skin lesions and immune dysregulation in pristane-induced murine lupus [58].

Despite all the caveats related to global modulation of immunometabolism in autoimmunity, there is also promising data in which systemic treatment with metformin (to attenuate mitochondrial function) together with 2-deoxyglucose (to reduce exacerbated glycolysis) displayed beneficial effects in murine lupus models and in vitro experiments with T cells from patients with SLE [59,60]. However, whether these potentially promising preclinical observations following manipulation of mitochondrial dysfunction and immunometabolism will be recapitulated in patients with SLE remains to be determined.

## Conclusion

Significant advances in understanding mitochondrial physiology and metabolism in immune cells highlight that targeting abnormalities in these pathways could benefit the treatment of autoimmune diseases. Indeed, future research should focus on developing strategies to generate effective immune tolerance, mitigate mitochondrial dysfunction, and enhance mitochondrial functionality. More research into metabolic dysfunction in various immune cell subsets in autoimmunity, its triggers, and cell-specific versus systemic effects will be needed before more targeted approaches can be designed. Efforts toward modifying aberrant mitochondrial physiology may help prevent or revert tissue damage in systemic inflammatory conditions. Given the complex effects of immune metabolism in homeostasis and disease, it remains unclear whether these approaches could also promote significant deleterious effects. It is expected that the next decade, with the significant advances in multi-omic approaches and disease pathophysiology will clarify the benefit, risks, and feasibility of targeting mitochondrial dysfunction in chronic autoimmune diseases.

## Abbreviations

APC: antigen-presenting cell
ATP: adenosine triphosphate
DAMP: danger-associated molecular pattern
DC: dendritic cell
IFN: interferon
ILC: innate lymphoid cell
LDG: low-density granulocytes
mDNA: mitochondrial DNA
MHC: major histocompatibility complex
mROS: mitochondrial reactive oxygen species
NAD: nicotinamide adenine dinucleotide
NADPH: nicotinamide adenine dinucleotide phosphate
NET: neutrophil extracellular trap
PRDX6: peroxiredoxin 6
RA: rheumatoid arthritis
ROS: reactive oxygen species
SLE: systemic lupus erythematosus
TCA: tricarboxylic acid
TLR: toll-like receptor
VDAC-1: voltage dependent anion channel-1
